# Effect of high-intensity intermittent rehabilitation training on physical function, gut microbiome and metabolite after percutaneous coronary intervention in patients with coronary heart disease

**DOI:** 10.3389/fcvm.2024.1508456

**Published:** 2024-11-28

**Authors:** Lei Jiang, Pu Liu, Mei Wang, Qiufeng Deng, Jiangpeng Wang, Yan Jiang, Ye Zhu, Haoyu Meng, Xiao Lu, Xiangqing Kong, Leilei Chen

**Affiliations:** ^1^ Department of Cardiology, The First Affiliated Hospital of Nanjing Medical University, Nanjing, China; ^2^ Department of Cardiology, Yili Friendship Hospital, Yili Kazak Autonomous Prefecture, Yili, China; ^3^ Department of Cardiology, The Fifth People’s Hospital of Huaian, Huaian, China; ^4^ Department of Rehabilitation, The First Affiliated Hospital of Nanjing Medical University, Nanjing, China

**Keywords:** gut microbiome, metabolite, high-intensity interval training, coronary heart disease, moderate-intensity continuous training

## Abstract

**Background:**

Postoperative rehabilitation exercise training after percutaneous coronary intervention (PCI) is crucial for coronary heart disease (CHD) patients in restoring health and preventing recurrence, including high-intensity interval training (HIIT). However, the impact of HIIT on cardiopulmonary function, gut microbiome and metabolite remains underexplored.

**Methods:**

This study included 60 patients with CHD who underwent percutaneous coronary intervention (PCI). Participants were divided into two groups: 33 in the moderate-intensity continuous training (MCT) group and 27 in the high-intensity interval training (HIIT) group. We assessed difference between two training in cardiopulmonary function, 6-minute walk test (6MWT) performance, biochemical indicators, plasma metabolites, and gut microbiome feature at baseline and after 3 months training. Furthermore, we analyzed 6MWT association to gut microbiome and metabolites with group differences.

**Results:**

The 6MWT showed significantly greater improvement in the HIIT group compared to the MCT group (*P* = 0.0024). Both groups showed reductions in low-density lipoprotein (LDL) levels and increases in peak oxygen uptake (VO2 peak) after training, but the HIIT group demonstrated a larger effect size in these measures. Moreover, subgroup analysis revealed that patients with a history of myocardial infarction (MI) in the HIIT group experienced a more substantial increase in VO2 peak compared to the MCT group (*P* = 0.04). In addition, we identified 29 gut microbial species and 30 plasma metabolites that were differentially enriched between the two groups, with some showing a significant impact on 6MWT performance.

**Conclusions:**

High-intensity interval training significantly improves 6MWT performance and exercise tolerance in cardiac rehabilitation patients, particularly enhancing VO2 peak in those with a history of MI. HIIT also appears to modulate the gut microbiome, increasing the abundance of *Clostridiales* and decreasing traumatic acid content, which may contribute to the observed improvements in exercise tolerance.

## Introduction

Coronary heart disease (CHD) is one of the leading causes of mortality worldwide, affecting over 110 million people each year and contributing to significant healthcare costs. In Europe alone, CHD is responsible for approximately 1.78 million deaths annually, while in the United States, it accounts for about 36% of all deaths (1–4). Similarly, CHD is also the major cause of death in China,the premature cardiovascular mortality rate will reach 11% by 2050 (5, 6).

Percutaneous coronary intervention (PCI) has emerged as a primary treatment for CHD, significantly alleviating symptoms and improving the quality of life for patients over the past few decades (7, 8).

Postoperative rehabilitation following PCI is crucial for restoring health and preventing recurrence, including pharmacological management, lifestyle modifications, and exercise rehabilitation. Some studies have shown that high-intensity interval training (HIIT) can enhance physical function after PCI (9, 10), others have demonstrated the benefits of moderate-intensity continuous training (MCT) for postoperative recovery (11, 12). However, the question of which exercise regimen is more effective for postoperative rehabilitation remains unanswered.

With advances in metabolomics and metagenomics, recent research has highlighted the close relationship between the gut microbiome, metabolites, and cardiovascular disease (13–17). Moreover, exercise has been shown to influence the abundance and metabolic capacity of the gut microbiome. For instance, Donati et al. reported that high-intensity exercise induced beneficial shifts in gut microbiome composition, promoting a healthier microbiome (18). Similarly, Denou et al. and Sun et al. found that HIIT increased gut microbiome diversity and metabolic capacity (19, 20). Despite this, there is limited knowledge about the diversity and composition of the gut microbiome and the alterations in plasma metabolites in CHD patients after PCI.

Given these findings, it is essential to investigate whether exercise training after PCI can modulate the gut microbiome and contribute to better postoperative outcomes. This study aims to explore the effects of a 12-week HIIT and MCT regimen on postoperative physical function, gut microbiome composition, and plasma metabolites in CHD patients after PCI, and to examine the role of specific gut microbiome and metabolites in postoperative recovery. The results will provide a theoretical basis for optimizing exercise regimens and considering gut microbiome-targeted therapies in CHD rehabilitation.

## Materials and methods

### Study design and participants

This clinical trial (NCT06575569) is a prospective, randomized, parallel, controlled study with blinded evaluation. It investigates the effects of high-intensity interval training (HIIT) on short-term cardiopulmonary rehabilitation, quality of life, and exercise tolerance gut microbiome composition and plasma metabolite in patients after percutaneous coronary intervention (PCI). A total of 60 patients with coronary heart disease (CHD) were recruited from the First Affiliated Hospital of Nanjing Medical University. All participants provided informed consent, and the study was approved by the Ethics Committee (2022-SR-425.A2).

### Selection criteria

Inclusion criteria were as follows: (1) Patients with CHD confirmed by coronary angiography, showing at least one coronary vessel with a stenosis greater than 70%. (2) Age between 18 and 70 years, with stable sinus rhythm. (3) Left ventricular ejection fraction (LVEF) greater than 40%. (4) Informed consent and voluntary participation.

Exclusion criteria included: (1) Severe organic cardiac or pulmonary diseases. (2) Physical movement disorders such as hemiplegia. (3) History of mental illness. (4) Uncontrolled hypertension or hemodynamic instability. (5) Severe nephropathy or peripheral artery disease. (6) Bone and joint diseases unsuitable for exercise. (7) Uncontrolled endocrine diseases. (8) Recent use of antibiotics or anti-diarrheal medications within the last 3 months.

### Study groups

We recruited 60 patients with more than 75% stenosis of major coronary arteries who had undergone coronary angiography. Participants were consecutively enrolled and randomly (random number table method) assigned to two groups: 27 patients (45.0%) to the HIIT group, including 15 with myocardial infarction (MI) and 12 without MI, and 33 patients (55.0%) to the moderate-intensity continuous training (MCT) group, including 13 with MI and 20 without MI. The median age of participants was 58 years, with 83% male. Baseline and post-intervention data were collected, including fecal, plasma samples, serum samples, cardiopulmonary function tests, 6-minute walk tests, and biochemical indicators.

### Cardiopulmonary function testing

Cardiopulmonary function was evaluated using a cardiopulmonary exercise function tester (model Smax58ce-sp, Nanjing, China). Patients sat on a stationary bicycle (model XRCISE CYCLE MED, Germany), warmed up for 5 min, and then rested for 3 min. They were fitted with monitoring devices for blood pressure and ECG. The initial power was set to 5W, with a rate of 10W/min, and patients were instructed to pedal at 50–60 rpm until symptoms such as dyspnea or chest pain occurred, at which point the exercise was terminated, and the peak power was recorded as the maximum exercise load.

### Exercise intervention programs

HIIT Group: After 2 weeks of adaptive training without adverse events, patients continued with intensive interval training under specialist supervision for 12 weeks. The program consisted of 30–40 min of exercise per session, including a 5-minute warm-up and a 5-minute cool-down period. The main exercise included four intervals of 4 min at 85%–95% of maximum heart rate (HR) reserve, followed by 3 min at 50%–70% of maximum HR reserve. This cycle was repeated four times. Patients were provided with exercise logs and encouraged to exercise regularly, supported by cardiovascular nurses who provided health education materials.

Subject maximum heart rate HRmax = 196.86−0.74 × age (21).

MCT Group: Patients in this group received routine postoperative care and follow-up. After 2 weeks of adaptive training, they participated in a 12-week exercise program consisting of 30–40 min per session, with intensity at 70%–75% of maximum HR reserve. Warm-up and cool-down periods included stretching, flexibility exercises, and low to medium intensity activities (50%–70% HR reserve). Patients were also given exercise logs and received health education materials.

### Metagenomic data generation and preprocessing

Participants were provided with swab tube to collect faecal samples both at baseline and follow-up for DNA sequencing and analysis. Once participants collected their faecal samples, samples were put into sealing bags with an ice pack to keep the samples frozen during transport back to the lab. All samples were stored at −80°C until analysis in the lab. Fecal DNA isolation was performed using the QIAamp Fast DNA Stool Mini Kit (Qiagen, cat. 51604). After DNA extraction, fecal DNA was used for library preparation, and whole-genome shotgun sequencing was performed on the Illumina NovaSeq-6000 plat- form (RRID:SCR_020150). From the raw metagenomic sequencing data, low-quality reads were discarded by the sequencing facility, and reads belonging to human contaminations were removed by mapping the data to the reference genomes using Bowtie2 (RRID:SCR _ 016368; v2.4.5). After filtering, on average, 54.8 million (SD = 14.7 million) paired reads per sample were obtained for subsequent analysis.

### Microbial taxonomies

Microbial taxonomic profiles were generated using MetaPhlAn4 (version 4.0.3). MetaPhlAn4 relies on nearly 5.1 million unique clade-specific marker genes identified from approximately 1M reference genomes, allowing unambiguous taxonomic assignments, accurate estimation of organismal abundance, and species-level resolution for bacteria, arc haea, eukaryotes, and viruses. 351 microbial species present in more than 20% of the samples were included for further analyses.

### Plasma untargeted metabolomics

2 ml of plasma was collected and stored at −80°C until analysis. During extraction, plasma samples were thawed on ice, vortexed and spun down. 20 mg plasma samples were mixed with 500 μl of 80% methanol and vortexed three times for 1 min. Then samples incubated at 4°C for 1 h to precipitate proteins and subsequently centrifuged at 14,000 g for 15 min.

The untargeted metabolome analysis of plasma samples was performed using an LC-MS/MS system comprising a ThermoFisher Vanquish UHPLC system coupled with an Orbitrap Q ExactiveTMHF mass spectrometer. The raw data files generated by UHPLC-MS/MS were processed using Compound Discoverer 3.1 (CD3.1, ThermoFisher) software for peak alignment, peak picking, and quantitation of each metabolite. The normalized data was used to predict the molecular formula based on additive ions, molecular ion peaks, and fragment ions. The identified peaks were matched with databases such as mzCloud, mzVault, and MassList to obtain accurate qualitative and relative quantitative results. In total, 763 metabolites were identified in the analyses. Metabolite annotations were performed using databases such as KEGG, HMDB, and LIPIDMaps.

### Statistical analysis

All statistical tests were performed using R (version 4.3.2). Patient characteristics were presented as medians (IQRs) or frequencies (proportions) for continuous and categorical variables, respectively. Group differences were assessed using the Wilcoxon rank-sum test for continuous variables and Pearson's chi-square or Fisher's exact test for categorical variables. Subgroup analyses were conducted to evaluate the effects of HIIT on phenotypes, particularly in patients with preoperative myocardial infarction. Separately, we used multivariable linear regression models to examine gut microbiome or metabolites (predictors) with phenotypes (outcomes), adjusting for age and sex (outcomes = predictor+age + sex). Statistical significance was defined as *p* < 0.05.

## Results

In this study, we recruited 60 patients diagnosed with coronary heart disease (CHD) via coronary angiography. The patients were randomly divided into two groups: a moderate-intensity continuous training (MCT) group (*n* = 33) and a high-intensity interval training (HIIT) group (*n* = 27). The baseline characteristics and clinical features of the participants are summarized in Table 1. Patients in the HIIT group were younger and more likely to be male, and other clinical features showed no significant differences between the two groups at baseline.

**Table 1:** Basic characteristics.

Variable	Overall, *N* = 60^a^	MCT, *N* = 33^a^	HIIT, *N* = 27^a^	*p*-value^b^
Gender				0.001
Female	10 (17%)	10 (30%)	0 (0%)	
Male	50 (83%)	23 (70%)	27 (100%)	
Age(years)	58 (51, 63)	59 (55, 64)	51 (47, 59)	0.003
MI				0.2
No	32 (53%)	20 (61%)	12 (44%)	
Yes	28 (47%)	13 (39%)	15 (56%)	
Stent_vessel				0.9
LAD	27 (45%)	13 (39%)	14 (52%)	
LAD and LCX	5 (8.3%)	2 (6.1%)	3 (11%)	
LAD, LCX and RCA	2 (3.3%)	2 (6.1%)	0 (0%)	
LAD and RCA	10 (17%)	5 (15%)	5 (19%)	
LCX	3 (5.0%)	2 (6.1%)	1 (3.7%)	
LCX and RCA	6 (10%)	4 (12%)	2 (7.4%)	
RCA	7 (12%)	5 (15%)	2 (7.4%)	
NT_PROBNP (pg/ml)	98 (60, 374)	170 (61, 410)	88 (60, 241)	0.2
Peak oxygen uptake (ml/kg/min)	13.5 (10.6, 15.9)	12.7 (10.1, 15.5)	14.5 (11.3, 18.0)	0.1
LVEF (%)	63.0 (60.8, 64.6)	63.0 (60.9, 65.2)	63.0 (60.8, 64.4)	0.7
LVIDs (mm)	32 (30, 34)	30 (29, 34)	32 (30, 35)	0.4
LVIDd (mm)	48 (45, 51)	48 (45, 51)	49 (45, 52)	0.3
LPWD (mm)	10.00 (9.00, 10.00)	10.00 (8.00, 10.00)	10.00 (10.00, 10.50)	0.084
TBIL (ummol/L)	12.2 (10.9, 14.5)	12.0 (9.9, 14.4)	12.3 (11.2, 15.4)	0.4
DBIL (ummol/L)	4.95 (3.65, 5.53)	4.90 (3.50, 6.00)	5.00 (3.75, 5.30)	0.8
r_GT (U/L)	20 (15, 26)	19 (15, 23)	22 (18, 29)	0.12
Creatinine (ummol/L)	72 (65, 82)	71 (64, 79)	75 (68, 85)	0.11
Urea (umol/L)	340 (286, 399)	339 (288, 384)	344 (267, 425)	0.5
Previous_history_hypertension				>0.9
No	33 (55%)	18 (55%)	15 (56%)	
Yes	27 (45%)	15 (45%)	12 (44%)	
Previous_history_diabetes				0.4
No	53 (88%)	28 (85%)	25 (93%)	
YES	7 (12%)	5 (15%)	2 (7.4%)	
Previous_history_smoking				0.051
No	37 (62%)	24 (73%)	13 (48%)	
Yes	23 (38%)	9 (27%)	14 (52%)	
Family history -CHD				0.2
No	57 (95%)	30 (91%)	27 (100%)	
Yes	3 (5.0%)	3 (9.1%)	0 (0%)	
Previous_use_betablockers				0.4
No	53 (88%)	28 (85%)	25 (93%)	
Yes	7 (12%)	5 (15%)	2 (7.4%)	
SBP (mmHg)	124 (117, 137)	124 (120, 137)	123 (116, 136)	0.5
DBP (mmHg)	80 (72, 86)	80 (72, 87)	76 (72, 86)	0.5
Heart_rate (bpm)	75 (68, 82)	75 (68, 85)	75 (69, 78)	0.5
Dyslipidaemic Agents				0.7
Atorvastatin	5 (8.3%)	2 (6.1%)	3 (11%)	
Atorvastatin and Ezetimibe	8 (13%)	3 (9.1%)	5 (19%)	
Atorvastatin and Aliciubicin	1 (1.7%)	1 (3.0%)	0 (0%)	
Rosuvastatin	13 (22%)	7 (21%)	6 (22%)	
Rosuvastatin and Ezetimibe	33 (55%)	20 (61%)	13 (48%)	
ACEI/ARB				0.3
No	31 (52%)	19 (58%)	12 (44%)	
Yes	29 (48%)	14 (42%)	15 (56%)	
Beta_blockers				0.7
No	27 (45%)	14 (42%)	13 (48%)	
Yes	33 (55%)	19 (58%)	14 (52%)	

ACEI, angiotensin-converting enzyme inhibitor; ARB, angiotensin-receptor blocker; MI, myocardial infarction; LAD, left ascending coronary artery; LCX, left circumflex coronary artery; RCA, right coronary artery; LVEF, left ventricular ejection fraction; LVID,left ventricular internal diameter; LPWD, left ventricular posterior wall; TBIL, total bilirubin; DBIL, direct bilirubin; r-GT, R-glutamyl transpeptidase; CHD, coronary heart disease; SBP, systolic blood pressure; DBP, diastolic blood pressure.

^a^ *n* (%); Median (IQR).

^b^ Fisher's exact test; Wilcoxon rank sum test; Pearson's Chi-squared test.

To assess the impact of different postoperative exercise training methods on recovery after PCI, we compared changes in peak oxygen uptake (VO2peak), 6-minute walk test (6MWT) performance, blood glucose levels, high-density lipoprotein (HDL), low-density lipoprotein (LDL), and lipoprotein(a) levels before and after the 3-month exercise intervention (Table 2). After adjusting for gender and age, the 6MWT showed significant improvement in the HIIT group (*P* = 0.0024). Additionally, both HIIT and MCT positively influenced LDL levels and VO2peak, with the HIIT group showing a greater reduction in LDL and a greater improvement in VO2peak, although the differences were not statistically significant.

**Table 2:** Phenotypic differences across all individuals.

Mean ± Sd	MCT			HIIT			Δ p_wilcox	Δ p_wilcox^a^
	Baseline	3-month	Δ	Baseline	3-month	Δ		
Steps_6minute (meter)	420.3 ± 63.7	507.9 ± 58.5	87.7 ± 26.8	469.4 ± 59.5	597 ± 48.7	127.6 ± 41.1	8.1 × 10-5	2.4 × 10-3
Initial_oxygen_uptake (ml/kg/min)	12.7 ± 3.5	18.9 ± 4.6	6.2 ± 4	14.5 ± 3.8	22.4 ± 3.6	7.8 ± 3.7	0.04	0.16
Initial_prob (%)	47.8 ± 12.7	67.8 ± 15.1	20 ± 12.1	49.2 ± 13.2	71.7 ± 10.6	22.5 ± 12.4	0.41	0.63
Glucose (mmol/L)	5.2 ± 1.6	5.7 ± 1.2	0.5 ± 1.9	5.1 ± 0.8	5.5 ± 1	0.3 ± 0.9	0.35	0.63
LDL (mmol/L)	2.1 ± 0.5	1.5 ± 0.4	-0.6 ± 0.6	2.6 ± 0.9	1.4 ± 0.4	-1.3 ± 1	3.7 × 10-3	0.1
HDL (mmol/L)	1 ± 0.2	1.1 ± 0.2	0.2 ± 0.2	1 ± 0.2	1.1 ± 0.1	0.1 ± 0.2	0.41	0.4
Lipoprotein_a (mg/L)	273.5 ± 318.3	329.3 ± 343.5	55.8 ± 129.7	292.7 ± 327.7	312.4 ± 317.6	19.8 ± 146.5	0.44	0.77

LDL, low density lipoprotein; HDL, high density lipoprotein; Initial_prob, Initial predicted percent value for cardiopulmonary test.

^a^ adjusted for gender and age.

In a subgroup analysis focusing on patients with myocardial infarction (MI), VO2peak was significantly better in the HIIT group compared to the MCT group (*P* = 0.04) after adjusting for gender and age. Besides, the HIIT group demonstrated greater reductions in LDL and improvements in 6MWT performance compared to the MCT group on patients with MI, although these differences were not statistically significant (Table 3).

**Table 3:** Phenotypic differences in individuals with MI.

Mean ± Sd	MCT			HIIT			Δ p_wilcox	Δ p_wilcox^a^
	Baseline	3-month	Δ	Baseline	3-month	Δ		
Steps_6minute (meter)	416 ± 61.5	506.5 ± 60.4	90.5 ± 24.6	467.7 ± 60	594.1 ± 53.8	121.3 ± 40.8	0.02	0.06
Initial_oxygen_uptake (ml/kg/min)	12.1 ± 3	17.1 ± 3.4	4.9 ± 2.4	14.5 ± 3.5	22.5 ± 3.8	8.3 ± 4.2	0.02	0.04
Initial_prob (%)	44.6 ± 13.3	61.9 ± 14.8	17.3 ± 9.6	48.8 ± 11.5	72.6 ± 11	24.5 ± 13.9	0.17	0.24
Glucose (mmol/L)	5.7 ± 1.9	5.7 ± 1	0 ± 2.1	4.9 ± 0.6	5.3 ± 0.8	0.3 ± 0.8	0.61	0.68
LDL (mmol/L)	2 ± 0.4	1.4 ± 0.3	−0.6 ± 0.4	2.5 ± 0.9	1.4 ± 0.5	−1.1 ± 1	0.23	0.68
HDL (mmol/L)	0.9 ± 0.2	1.1 ± 0.2	0.3 ± 0.3	0.9 ± 0.2	1.1 ± 0.1	0.1 ± 0.2	0.26	0.29
Lipoprotein_a (mg/L)	387.8 ± 390.5	432.6 ± 430.6	44.9 ± 154.6	303.5 ± 339.6	304.8 ± 347.5	15.7 ± 176.2	0.87	0.89

LDL, low density lipoprotein; HDL, high density lipoprotein; Initial_prob, Initial predicted percent value for cardiopulmonary test.

^a^ adjusted for gender and age.

**Figure 1:**
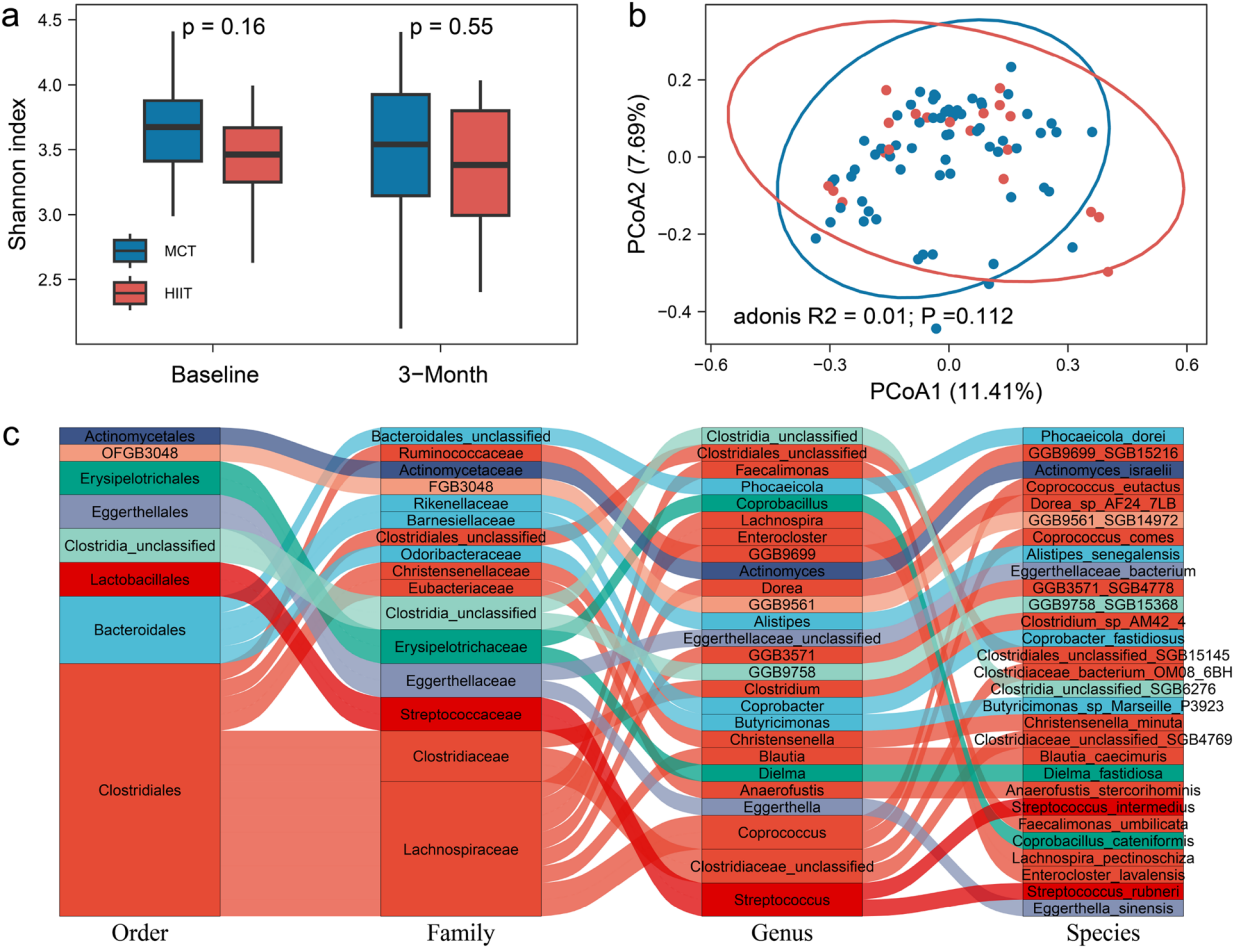
**(a)** The alpha diversity at the species level between the exercise groups at both baseline and after 3 months of training. **(b)** Principal coordinate analysis (PCoA) of the gut microbiome profiles in microbiome composition between the HIIT and MCT groups. **(c)** 29 species between the groups after 3 months of training.

Furthermore, we examined the effects of different exercise training methods on gut microbiome feature and plasma metabolites. Exercise training increases the relative abundance of the butyrate-producing taxa. Butyrate is the SCFA produced by the fermentation of dietary fiber bacteria. Butyrate, as the main fuel of colon cells, can promote the proliferation of colonic epithelial cells, promote the integrity of the intestinal barrier, and regulate the host immune system and gene expression.The alpha diversity at the species level showed no significant differences between the exercise groups at both baseline and after 3 months of training (P_baseline_ = 0.16, P_3−month_ = 0.55, Figure 1a). Principal coordinate analysis (PCoA) of the gut microbiome profiles also showed no significant shifts in microbiome composition between the HIIT and MCT groups (*P* = 0.11, Figure 1b). Next, we further tested group difference of the relative abundance of 351 gut microbial species. Our results showed 29 species exhibited significant differences between the groups after 3 months of training, and they did not differ at baseline (Figure 1c, Supplementary Table S1). These species included *Actinomyces_israelii*, *Alistipes_senegalensis*, *Anaerofustis_stercorihominis*, *Blautia_caecimuris*, *Butyricimonas_sp_Marseille_P3923*, *vi Christensenella_minuta*, *Clostridia_unclassified_SGB6276*, *Clostridiaceae_bacterium_OM08_6BH*, *Clostridiaceae_unclassified_SGB4769*, *Clostridiales_unclassified_SGB15145*, *Clostridium_sp_AM42_4*, *Coprobacillus_cateniformis*, *Coprobacter_fastidiosus*, *Coprococcus_comes*, *Coprococcus_eutactus*, *Dielma_fastidiosa*, *Dorea_sp_AF24_7LB*, *Eggerthella_sinensis*, *Eggerthellaceae_bacterium*, *Enterocloster_lavalensis*, *Faecalimonas_umbilicata*, *GGB3571_SGB4778*, *GGB9561_SGB14972*, *GGB9699_SGB15216*, *GGB9758_SGB15368*, *Lachnospira_pectinoschiza*, *Phocaeicola_dorei*, *Streptococcus_intermedius*, *Streptococcus_rubneri Intestinal*, with 16 of these belonging to the *Clostridiales* order, which has been linked to exercise in previous studies (22, 23).

**Figure 2:**
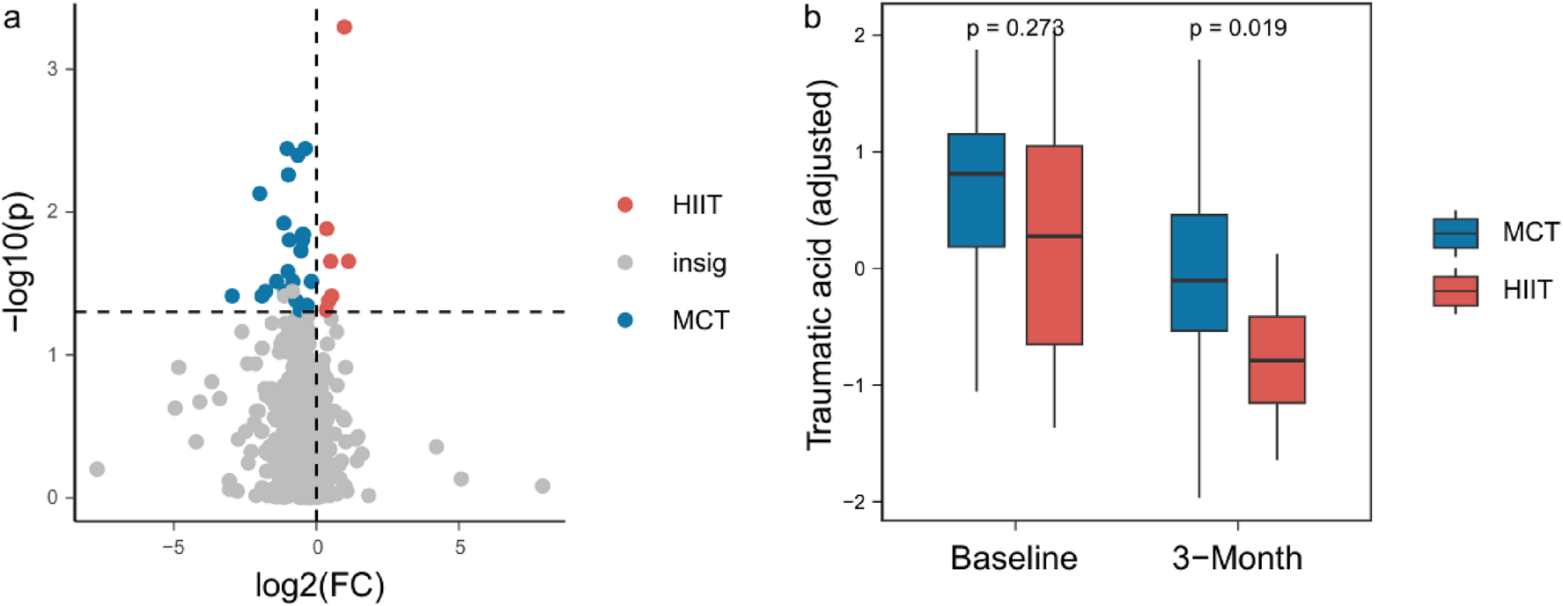
**(a)** 30 plasma metabolites between the groups after 3 months of training. **(b)** Traumatic acid levels between the two groups in baseline and after the 3-month intervention.

Additionally, we identified 30 plasma metabolites that showed significant differences between the groups after 3 months of training, and not at baseline (Figure 2a, Supplementary Table S2). For example, traumatic acid levels did not differ between the two groups in baseline (*P* = 0.273), while they were significantly lower in the HIIT group compared to the MCT group after the 3-month intervention (*P* = 0.019, Figure 2b).

**Figure 3:**
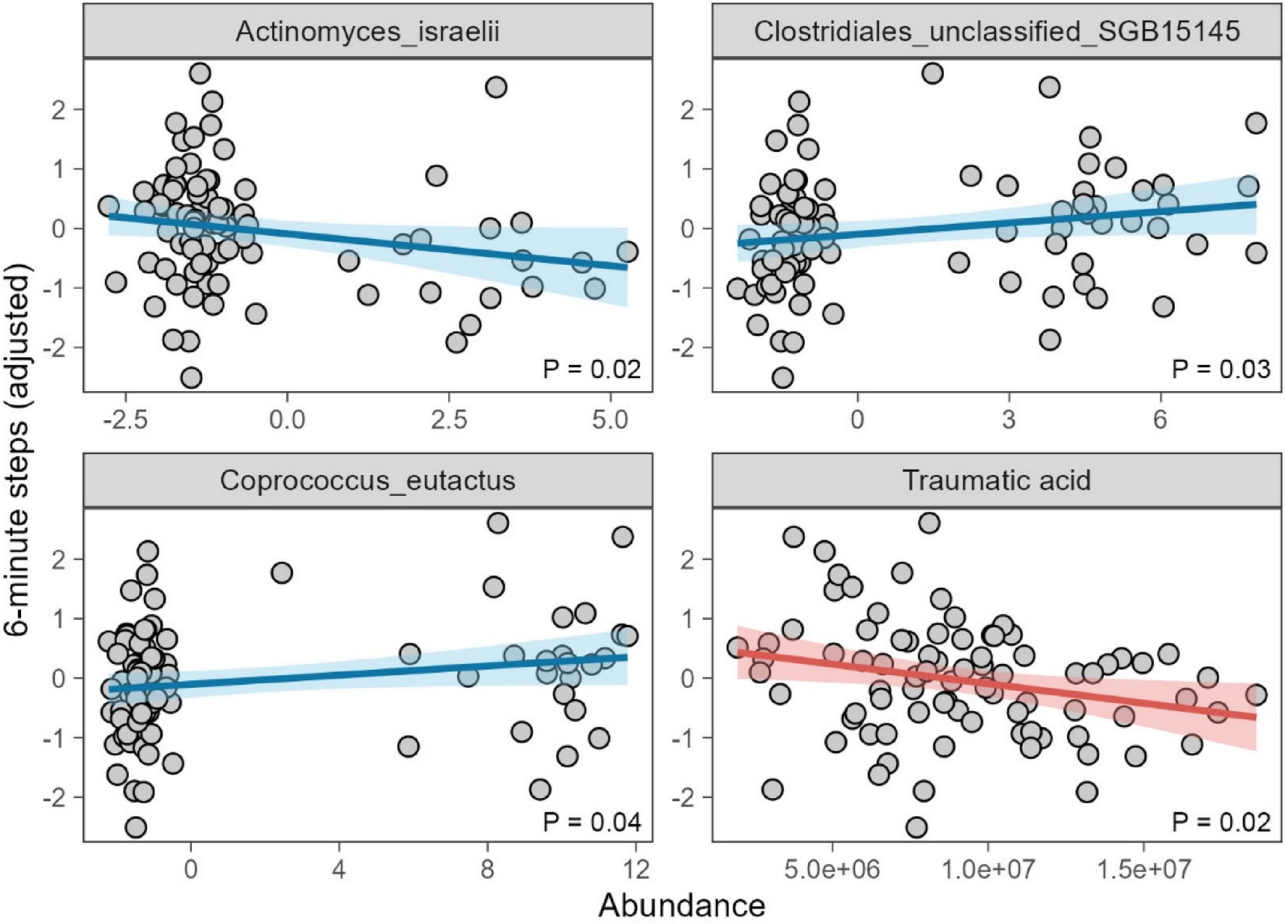
6 minite steps association to *Actinomyces israelii*, *Coprococcus eutactus*, and *Clostridiales unclassified SGB15145* adjusted gender and age using liner model.

To determine whether these gut microbiome changes or metabolite alterations influenced postoperative rehabilitation outcomes, we conducted correlation analyses between the 29 identified microbial species, the 30 metabolites, and the 6MWT performance (Supplementary Table S3, S4). After adjusting for gender and age, our results revealed that *Actinomyces israelii*, *Coprococcus eutactus*, and *Clostridiales unclassified SGB15145,* and traumatic acid significantly impacted 6MWT performance, suggesting that specific gut microbial species and metabolites may influence exercise tolerance and rehabilitation outcomes following PCI (Figure 3).

## Discussion

Coronary heart disease (CHD) is one of the most common chronic cardiovascular conditions. While percutaneous coronary intervention (PCI) effectively improve myocardial perfusion and alleviate clinical symptoms, it do not reverse the underlying pathophysiological changes associated with the disease (24). Exercise rehabilitation is increasingly recognized as a crucial strategy for improving outcomes in patients with cardiovascular disease (25–27). Researches have demonstrated that different types of exercise training, including high-intensity interval training (HIIT) and the moderate-intensity continuous training (MCT), can improve postoperative rehabilitation of CHD patients after PCI (28–30). A large number of certificates shown that VO2 peak is known for future cardiovascular event and mortality force predictor, even a small increase in VO2 peak leads to all the risk of cause-related and cause-specific mortality is greatly reduced (31–33).

Our results suggested HIIT significantly enhanced key cardiopulmonary indicators compared to MCT, including VO2 peak and 6-minute walk test performance in patients with CHD, especially those with a history of myocardial infarction. Postoperative rehabilitation exercise training improves endothelial cell regeneration in injured vessels and reduces chronic inflammation by increasing the number of vascular endothelial progenitor cells (34–36). Schmid et al. (37) observed an increase in endothelial progenitor cells following exercise, while other research indicates that high-intensity interval training (HIIT) significantly reduces white blood cell count, red blood cell count, hemoglobin percentage, and hematocrit values, thereby improving the inflammatory response in the vascular endothelium (38). Additionally, HIIT has been shown to enhance the responsiveness of smooth muscle cells (39). Kramann et al. (40) and Evrard et al. (41) demonstrated that adventitial fibroblast-like cells are functionally implicated in plaque ECM production, and aerobic exercise may regulate fibroblast inflammasome-mediated pyroptosis to inhibit atherogenesis (42). Wang et al. (43) found that HIIT reduces the expression of macrophages and M1 macrophages by lowering the mRNA levels of inflammatory factors (TNF-α, IL-6, and MCP-1) in mice.

HIIT also has an effect on the gut microbiome of CHD patients after PCI. Our study revealed that patients high-intensity interval training enriches more beneficial bacteria compared to the moderate-intensity continuous training after PCI. Specific gut microbiomes improved 6MWT, including Coprococcus eutactus and Clostridiales unclassified SGB15145 species. These gut microbiome plays a role in regulating endothelial cells via toll-like receptors (TLR 2, 4) to inhibit inflammatory markers by reducing lipopolysaccharides (LPSs). Reducing systemic inflammation can slow the progression of atherosclerosis, prevent the formation of vulnerable areas, and inhibit immune system activation (44–47). Research suggests that these gut microbiomes can reduce leukocyte infiltration and modulate the immune response (48–50). Furthermore, abnormal phenotypic switching of VSMCs in vessels is markedly alleviated by these gut microbiomes and its metabolite butyrate (49).

Traumatic acid, identified as a potential plasma biomarker for sarcopenia, has been negatively associated with this condition. Plasma traumatic acid concentrations show a significant decline across groups, from elderly subjects with sarcopenia to those without, and to younger adults (51). It is well established that exercise tolerance is positively correlated with muscle mass, and active exercise can increase muscle mass. Several studies have concluded that the pathophysiology of sarcopenia is linked to the production of pro-inflammatory cytokines, prostaglandins, and chronic inflammation (52, 53). As a plant wound hormone, traumatic acid is an intermediate in prostaglandin synthesis and plays a crucial role in maintaining stable physiological functions and regulating skeletal muscle (52, 54–56). Lower levels of traumatic acid are associated with reduced muscle mass, strength, and gait speed. This is consistent with our findings that HIIT improved the 6-minute walking distance. We propose that traumatic acid could be a potential marker for exercise tolerance.This study discusses the influence of different intensity exercise on the cardiopulmonary function of patients, and revealing the exercise can change the intestinal microbial and metabolic indicators, and preliminary discusses the reasons behind the exercise can change the cardiopulmonary function, for the future research provides some theoretical evidence of exercise and cardiopulmonary function mechanism, and provide new ideas for clinical improvement of cardiopulmonary function. In this regard, further and more detailed studies on the specific modifications produced by physical activity on the microbiota composition could be useful to explore new approaches for the treatment of metabolic and inflammatory diseases in which the microbiota plays a fundamental role.

However, our study also has some limitations, including a small sample size, short follow-up, and uneven gender distribution. With a sample size of 60 participants, our study may lack sufficient statistical power to detect subtle or small-effect changes in the gut microbiome, limiting the robustness of our conclusions. Future research with a larger sample size would improve statistical power, enhance the reliability of results, and allow for more definitive conclusions regarding the observed effects. The follow-up period in our study was relatively short, which may not fully capture the long-term effects of interventions on the gut microbiome. Microbiome changes often require more extended periods to stabilize and reveal meaningful trends. Thus, our findings primarily reflect short-term responses and may not predict long-term outcomes. Longer follow-up periods in future studies would provide a more comprehensive understanding of the sustained effects of the interventions on gut microbiome composition and diversity. The study's uneven gender distribution, particularly the absence of female participants in the HIIT group, introduces potential bias. Gender differences in both physiological responses and microbiome composition have been documented, and the lack of female representation in certain groups limits the applicability of the findings to a mixed-gender population. This imbalance may also mask or exaggerate gender-specific effects, which could influence the overall conclusions. To improve generalizability, future studies should ensure balanced gender representation across all groups, allowing for a more accurate assessment of the intervention's effects across genders. In summary, while our findings contribute valuable insights, these limitations should be considered when interpreting the results. Future studies addressing these issues will provide a more comprehensive and generalizable understanding of the gut microbiome's response to the studied interventions.

## Conclusion

In summary, our study suggested that high-intensity interval training (HIIT) can significantly improve the 6-minute walk test (6MWT) and exercise tolerance compared to the moderate-intensity continuous training (MCT) in patients after percutaneous coronary intervention (PCI). Additionally, HIIT also positively impacts gut microbiome and plasma metabolite, some of which may help improve 6MWT and VO2peak, like Coprococcus eutactus and Clostridiales unclassified SGB15145 species. The results provide a theoretical basis for optimizing exercise regimens and considering gut microbiome-targeted therapies in CHD rehabilitation.

## Data Availability

The datasets presented in this study can be found in online repositories. The names of the repository/repositories and accession number(s) can be found in the article/Supplementary Material.
